# Ferroelectric properties of PZT/BFO multilayer thin films prepared using the sol-gel method

**DOI:** 10.1186/1556-276X-7-54

**Published:** 2012-01-05

**Authors:** Seo-Hyeon Jo, Sung-Gap Lee, Young-Hie Lee

**Affiliations:** 1 Department of Ceramic Engineering, Engineering Research Institute, Gyeongsang National University, Jinju-Si, 660-701, South Korea; 2 Department of Electronic Materials Engineering, Kwangwoon University, Seoul, 139-701, South Korea

## Abstract

In this study, Pb(Zr_0.52_Ti_0.48_)O_3_/BiFeO_3 _[PZT/BFO] multilayer thin films were fabricated using the spin-coating method on a Pt(200 nm)/Ti(10 nm)/SiO_2_(100 nm)/p-Si(100) substrate alternately using BFO and PZT metal alkoxide solutions. The coating-and-heating procedure was repeated several times to form the multilayer thin films. All PZT/BFO multilayer thin films show a void-free, uniform grain structure without the presence of rosette structures. The relative dielectric constant and dielectric loss of the six-coated PZT/BFO [PZT/BFO-6] thin film were approximately 405 and 0.03%, respectively. As the number of coatings increased, the remanent polarization and coercive field increased. The values for the BFO-6 multilayer thin film were 41.3 C/cm^2 ^and 15.1 MV/cm, respectively. The leakage current density of the BFO-6 multilayer thin film at 5 V was 2.52 × 10^-7 ^A/cm^2^.

## Introduction

Multiferroic materials, which exhibit simultaneously ferroelectric, ferromagnetic, antiferromagnetic, and ferroelastic behaviors, provide opportunities for potential applications in information storage, spintronic devices, and sensors [1]. Bismuth ferrite (BiFeO_3_) [BFO] is one such multiferroic material. BFO exhibits a distorted perovskite structure with rhombohedral symmetry. It belongs to the R3c space group with a unit cell parameter *a *= 0.5643 nm and *a *= 59.348° [2]. One of the striking features of BFO materials is the coexistence of ferroelectric (*T*_c _= 1,123 K) and antiferromagnetic orderings (*T*_N _= 643 K) at room temperature due to a residual moment from a canted spin structure [3]. BFO is attracting great attention as a promising ferroelectric material for high-density FeREMs because of its large remanent polarization. However, BFO has serious problems as a ferroelectric material, having quite a large leakage current density, especially at room temperature. Therefore, dielectric breakdown occurs easily even at a low field, thereby indicating the difficulty in poling films. Furthermore, the highly electrically conductive nature of BFO makes it difficult to obtain excellent ferroelectric properties. To overcome this problem, various approaches have been proposed, including a substitution technique using Mn, Ti at the B-site, and/or La and Nd at the A-site [4,5] and the formation of a solid solution with Pb(Zr, Ti)O_3 _and BaTiO_3 _compositions [6]. There are many reports on the reduction of the leakage current induced by doping and the formation of a solid solution. In these investigations, the capacitor structure formed from the metal-insulator-metal structure is used for current measurement. It should be noted that current measured in the capacitor structure includes contributions of the grain boundaries or a microstructure of the films. We have already reported on the good dielectric properties, especially the high remanent polarization and low leakage current densities of Pb(Zr_0.52_Ti_0.48_)O_3 _[PZT] heterolayered thin films, which were alternately spin-coated using Pb(Zr_0.20_Ti_0.80_)O_3 _and Pb(Zr_0.80_Ti_0.20_)O_3 _metal alkoxide solutions [7]. In this study, BFO/PZT multilayer thin films were prepared using the sol-gel method, which were spin-coated on the Pt/Ti/SiO_2_/Si substrate alternately using BFO and PZT metal alkoxide solutions. We also investigated the structural and dielectric properties of BFO/PZT multilayer thin films for application in electronic memory devices.

## Experimental details

Using the sol-gel method, BFO and PZT with excess Pb-acetate 10 mol% precursor solutions were prepared from the starting materials Bi-nitrate pentahydrate [Bi(NO_3_)_3_·5H_2_O], Fe-nitrate nonahydrate [Fe(NO_3_)_3_·9H_2_O], Pb-acetate trihydrate [Pb(CH_3_CO_2_)_2_·3H_2_O], Zr *n*-propoxide [Zr(OCH_2_CH_2_CH_3_)_4_], and Ti iso-propoxide {Ti[OCH(CH_3_)_2_]_4_}, and the solvent 2-methoxyethanol. The PZT precursor solution was passed through a syringe filter and spin-coated on the Pt(200 nm)/Ti(10 nm)/SiO_2_(100 nm)/p-Si(100) substrates using a spinner operated at 3,000 rpm for 20 s to form the first layer. These PZT films were dried at 573 K for 30 min to remove the organic materials and sintered at 873 K for 30 min to crystallize them into a perovskite structure. The BFO precursor solution was then spin-coated and dried on the PZT films under the same conditions and sintered at 873 K for 10 min to form the second BFO layer. This procedure was repeated several times, fabricating BFO/PZT multilayer thin films. The crystalline structure of the BFO/PZT multilayer films was analyzed by X-ray diffraction [XRD], and surface and cross-sectional morphologies of the films were examined by scanning electron microscopy [SEM]. To measure the ferroelectric properties, Pt films were dc sputter-deposited on the BFO/PZT films as the top electrode with a diameter of 200 μm. The leakage current and polarization-electric field [P-E] hysteresis loops were analyzed using a ferroelectric test system (RT66B; Radiant Technologies, Inc., Albuquerque, NM, USA).

## Results and discussion

**Figure 1:**
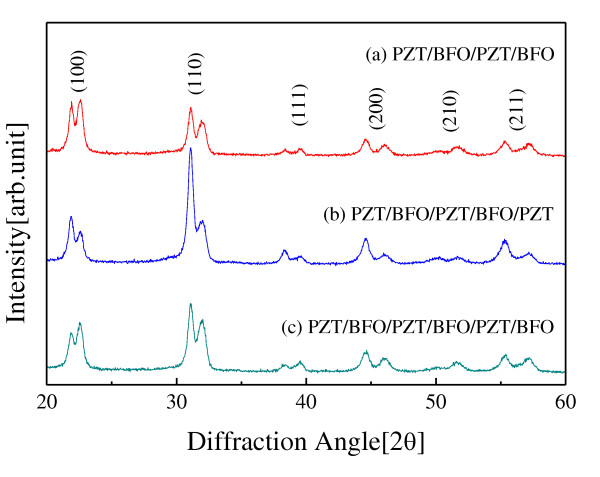
**XRD patterns of PZT/BFO multilayer thin films**.

Figure 1a, b, c shows the XRD patterns of PZT/BFO multilayer thin films. The XRD pattern was investigated through the GI-XRD method. All films showed the typical XRD patterns of a perovskite polycrystalline structure, and the second phase such as Bi_2_Fe_4_O_3 _or the preferred orientation was not observed. Generally, XRD patterns of PZT(52/48) thin films show a single peak for each diffraction angle. In addition, XRD patterns of BFO thin films show a single peak at 2*θ *= 22.5° and 46°. However, all PZT/BFO multilayer thin films showed that the XRD peak splits at each diffraction angle. This property may be understood in terms of the effect of the lower layer. The crystal growth of the upper BFO (or PZT) layer can be influenced by the lower PZT (or BFO) layers, and the crystallization behavior of the resultant film is controlled by choosing the initial layer or seeding layer.

**Figure 2:**
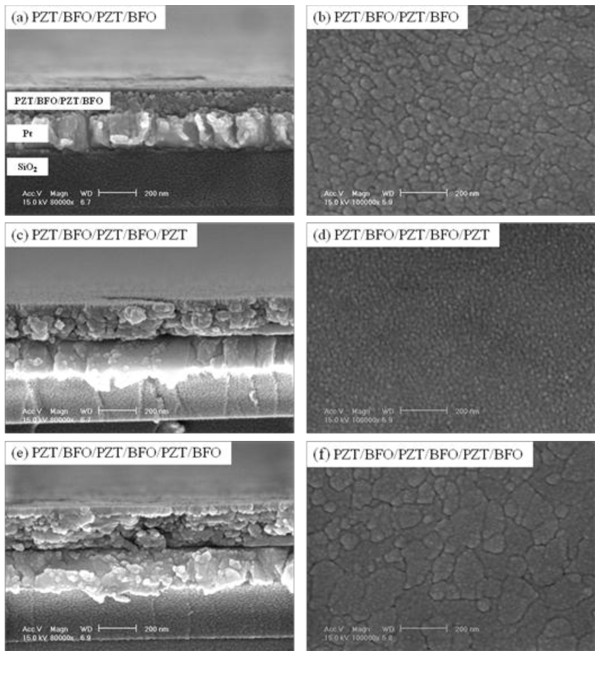
**SEM micrographs of PZT/BFO multilayer thin films**. Cross section of the (**a**) PZT/BFO/PZT/BFO, (**b**) PZT/BFO/PZT/BFO/PZT, and (**c**) PZT/BFO/PZT/BFO/PZT/BFO films and surface morphologies of the (**d**) PZT/BFO/PZT/BFO, (**e**) PZT/BFO/PZT/BFO/PZT, and (**f**) PZT/BFO/PZT/BFO/PZT/BFO films.

Figure 2 shows the cross section and surface SEM micrographs of PZT/BFO multilayer thin films. The average thickness of the film after one cycle of drying/sintering was approximately 45 to 55 nm, and the thickness of the PZT/BFO-6 film was 238 nm. All films consist of a fine grain structure with a relatively flat surface morphology, as shown in Figure 2a, b, c. PZT/BFO-4 and 6 films with a top layer of BFO shown in Figure 2d, f showed a dense grain structure. On the other hand, the PZT/BFO-5 film with a top layer of PZT shown in Figure 2e showed a fine and void-free grain structure. The average grain size increased with an increase in the number of coatings due to the increased number of heat treatments. The average grain sizes of the PZT/BFO-4 and 6 films were about 94 and 137 nm, respectively.

**Figure 3:**
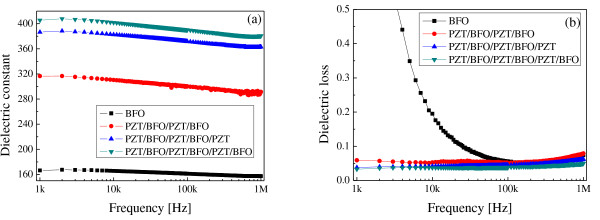
**Dielectric constant and dielectric loss of PZT/BFO multilayer thin films**. (**a**) Dielectric constant and (**b**) dielectric loss of PZT/BFO multilayer thin films as a function of applied frequency.

Figure 3 shows the dielectric constant and dielectric loss of the PZT/BFO multilayer thin films as a function of the measuring frequency from 1 kHz to 1 MHz. The relative dielectric constant decreased with an increase in the applied frequency, and the PZT/BFO multilayer thin films showed a typical frequency-dispersion property. The dielectric constant increased and the dielectric loss decreased with an increase in the number of coatings and an increase in the film thickness, and the PZT/BFO-6 film displays good results of 405 and 0.033%, respectively, at 1 kHz. PZT/BFO multilayer thin films exhibit a superior dielectric constant compared with a single-composition BFO film (166 at 1 kHz). According to a report by Wang et al. [1], the crystal structure of rhombohedral BFO thin films fabricated on a SrRuO_3 _[SRO] electrode changed into a monoclinic crystal structure due to the compressive stress imposed by the SRO electrode, which has an in-plane lattice parameter smaller than that of BFO. Furthermore, the magnitude of the ionic displacement relative to the centrosymmetric-strained perovskite structure was found to be extremely large. We believe that the rhombohedral crystal structure of the BFO film (*a *= 0.5634 nm) was distorted due to the large lattice mismatch and compressive stress imposed by the lower PZT(52/48) ceramic (*a *= 0.403 nm, *c *= 0.406 nm [8]). Therefore, the PZT/BFO multilayer thin film displayed good dielectric properties due to the large ionic displacements in the distorted perovskite structure. Dielectric loss decreased with an increase in the number of coatings. This phenomenon can probably be explained by the fact that the diffusion of Pb from the PZT film into the Pt bottom electrode [9] and the diffusion of Pb, Ti, Zr, Bi, and Fe at the interfaces of the PZT film and BFO film increase with an increase in the number of coatings, in other words, an increase in the number of annealing processes [7]. Therefore, interface layers formed between the PZT and BFO layers act as trap centers for the charges. However, further investigation and discussion are necessary to understand the dielectric properties of PZT/BFO multilayer films.

**Figure 4:**
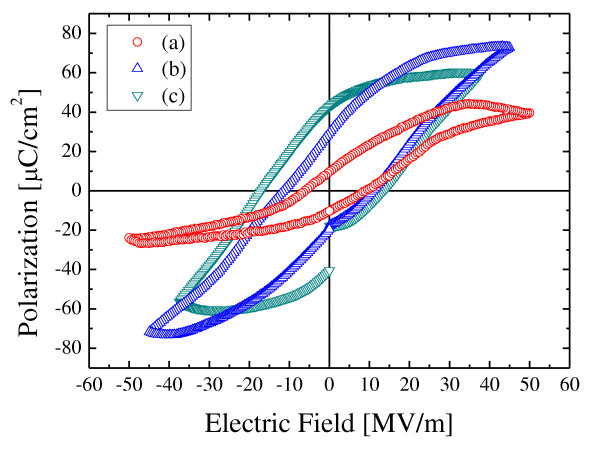
**P-E hysteresis loops of PZT/BFO multilayer thin films**. (**a**) PZT/BFO/PZT/BFO, (**b**) PZT/BFO/PZT/BFO/PZT, and (**c**) PZT/BFO/PZT/BFO/PZT/BFO films.

Figure 4 shows the hysteresis loops of PZT/BFO multilayer thin films. Well-saturated hysteresis loops could be obtained for all films. The remanent polarization and coercive field increased with an increase in the number of coatings. These properties can be understood in terms of the effect of grain size and the large ionic displacement in the distorted perovskite structure as discussed in Figure 3. Generally, by increasing film thickness, the stress induced from the substrate is reduced. However, in this study, despite an increase in film thickness, the coercive field increased with an increase in the number of coatings. This property can be understood in terms of the effect of interface layers formed between the PZT and BFO layers. The space-charge layer, which forms at the interfacial layer due to the diffusion of Pb, Ti, Zr, Bi, and Fe at the interfaces of the PZT and BFO films, acts to suppress the polarization rotation. The PZT/BFO-6 multilayer film shows a remanent polarization of 41.3 μC/cm^2 ^and a coercive field of 15.1 MV/m.

**Figure 5:**
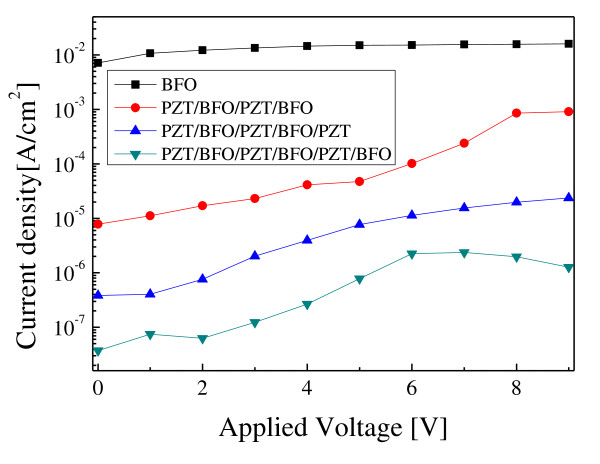
**Leakage current density characteristics with an applied voltage for PZT/BFO multilayer thin films**.

Figure 5 shows the leakage current densities of PZT/BFO multilayer thin films with the applied voltage. Leakage current densities of PZT/BFO multilayer thin films decreased with an increase in the number of coatings, and these are much lower values than a pure BFO thin film [5]. These results suggest that the oxygen vacancies were greatly reduced, and the trap centers of carriers were formed at the interfaces between the BFO and PZT films, which increased with an increase in the number of coatings. The leakage current density of the PZT/BFO-6 multilayer thin film is less than 2.5 × 10^-7 ^A/cm^2 ^at 5 V. This value can be applied to memory devices. However, further investigation and discussion are necessary to understand the leakage current mechanism in PZT/BFO multilayer thin films.

## Conclusions

In this study, PZT/BFO multilayer thin films were prepared using the sol-gel method, which were spin-coated on a Pt/Ti/SiO_2_/Si substrate alternately using PZT(52/48) and BFO alkoxide solutions. The average thickness of a film after one cycle of drying/sintering was approximately 45 to 55 nm. All PZT/BFO multilayer thin films show a dense and homogeneous grain structure with a relatively flat surface morphology. The dielectric properties such as dielectric constant, dielectric loss, remanent polarization, and leakage current density of PZT/BFO multilayer thin films were superior to those of single-composition BFO films, and those values for the PZT/BFO-6 film were 405, 0.033%, 41.3 μC/cm^2 ^and 2.5 × 10^-7 ^A/cm^2^, respectively, at 5 V. We believe that these properties of PZT/BFO multilayer films were caused by interface effects between the PZT and BFO films.

## Competing interests

The authors declare that they have no competing interests.

## Authors' contributions

S-HJ carried out the experiments and measurements. S-GL carried out the manuscript. Y-HL participated in the measurement. All authors read and approved the final manuscript.

## References

[B1] Wang J Neaton JB Zheng H Nagarajan V Ogale SB Liu B Viehland D Vaithyanathan V Schlom DG Waghmare UV Spaldin NA Rabe KM Wuttig M Ramesh R Epitaxial BiFeO_3 _multiferroic thin film heterostructures Science 2003 299 1719 10.1126/science.1080615 12637741

[B2] Yakoviev S Zekonyte J Solterbeck CH Es Souni M Interfacial effects on the electrical properties of multiferroic BiFeO_3_/Pt/Si thin film heterostructures Thin Solid Films 2005 493 24 10.1016/j.tsf.2005.06.020

[B3] Zheng RY Sim CH Wang J Effects of SRO buffer layer on multiferroic BiFeO_3 _thin films J Am Ceram Soc 2008 91 3240 10.1111/j.1551-2916.2008.02536.x

[B4] Singh SK Ishiwara H Sato K Maruyama K Microstructure and frequency dependent electrical properties of Mn-substituted BiFeO_3 _thin films J Appl Phys 2007 102 094109 10.1063/1.2812594

[B5] Kawae T Terauchi Y Tsuda H Kumeda M Morimoto A Improved leakage and ferroelectric properties of Mn and Ti codoped BiFeO_3 _thin films Appl Phys Lett 2009 94 112904 10.1063/1.3098408

[B6] Kim JK Kim SS Kim WJ Bhalla AS Guo R Enhanced ferroelectric properties of Cr-doped BiFeO_3 _thin films grown by chemical solution deposition Appl Phys Lett 2006 88 132901 10.1063/1.2189453

[B7] Lee SG Kim KT Lee YH Characterization of lead zirconate titanate heterolayered thin films prepared on Pt/Ti/SiO_2_/Si substrate by the sol-gel method Thin Solid Films 2000 372 45 10.1016/S0040-6090(00)01030-0

[B8] Randall CA Kim N Kucera JR Cao W Shrout TR Intrinsic and extrinsic size effects in fine-grained morphotropic-phase-boundary lead zirconate titanate ceramics J Am Ceram Soc 1998 81 677 688

[B9] Shen Z Chen Z Lu Q Qiu Z Jiang A Qu X Chen Y Liu R Nano-embossing technology on ferroelectric thin film Pb(Zr_0.3_, Ti_0.7_)O_3 _for multi-bit storage application Nano Res Lett 2011 6 474 10.1186/1556-276X-6-474 PMC3211987 21794156

